# Microbiological Quality and Antibiotic Resistance of Relevant Bacteria from Horsemeat

**DOI:** 10.3390/microorganisms12091775

**Published:** 2024-08-28

**Authors:** Elena Gonzalez-Fandos, Jessica da Silva Guedes

**Affiliations:** Food Technology Department, CIVA Research Center, University of La Rioja, Madre de Dios 53, 26006 Logroño, La Rioja, Spain

**Keywords:** horsemeat, antimicrobial resistance, food safety, staphylococci, *Staphylococcus delphini*, *Listeria monocytogenes*, *Yersinia enterocolitica*, *Vagococcus fluvialis*, *Stenotrophomonas maltophilia*, meat

## Abstract

The aim of this work was to assess the microbiological safety and quality of horsemeat. A total of 19 fresh horsemeat samples were analysed. Mesophile counts were 4.89 ± 1.08 log CFU/g, and *Enterobacteriaceae*, *Staphylococcus* spp., and enterococci were only isolated from 36.84%, 21.05%, and 15.79% of the samples, respectively. Neither *Staphylococcus aureus* nor *Escherichia coli* were found in any sample. *Listeria* spp. and *Listeria monocytogenes* were detected in 31.58% and 21.05% of the samples, respectively. *Campylobacter jejuni* was not detected in any sample. The dominant bacteria were lactic acid bacteria. Seven different *Staphylococcus* spp. were identified, the most common being *S. delphini*, *S. saprophyticus*, and *S. warneri*. *S. delphini* showed resistance against mupirocin and cefoxitin. All the *L. monocytogenes* strains showed resistance against ampicillin, cefotaxime, and oxacillin. Multi-resistant *Yersinia enterocolitica*, *Stenotrophomonas maltophilia*, and *Vagococcus. fluvialis* strains were found, with resistance to 11, 7, and 8 antibiotics, respectively, causing significant concern. Therefore, specific actions should be taken to decrease the contamination of horsemeat.

## 1. Introduction

Horsemeat has a high protein content, high levels of n-3 fatty acids, and it is a good source of minerals and vitamins [1,2]. Moreover, horsemeat is low fat and has a high unsaturated fatty acid content, as well as a high iron content compared with that of other meats, and as such, it is considered as a healthy alternative to other meats [1]. Due to its high iron content, it is of interest for use in the treatment of human iron-deficiency anaemia diseases [3]. Furthermore, horsemeat has attractive sensory characteristics [2]. However, the consumption of horsemeat is limited, as it is affected by cultural and ethical biases [1]. The highest consumption of horsemeat is found in countries in Central Asia, some countries in Europe and Mexico [4]. In 2020, 0.8 million tonnes of horsemeat were produced worldwide [4]. In the European Union, Spain is one of the largest producers of horsemeat, followed by Poland and Italy [4]. In 2022, the production of horsemeat in Spain amounted to 8246 tonnes [5].

Since horsemeat is not a common food in many countries, studies on the microbiological quality and safety of this type of meat are scarce [6,7,8]. Most of the studies are focused on lactic acid bacteria, pseudomonas, and *Enterobacteriaceae* [6,7,8,9]. Pathogenic bacteria such as *Escherichia coli* and *Listeria monocytogenes* can be present in horsemeat [9,10,11]. Since *Yersinia enterocolitica* and *Staphylococcus aureus* have often been found in horses, contamination of meat can occur during processing, depending on the hygienic conditions of the processing plant [10,12,13].

Since food-borne bacteria that are resistant to antibiotics could have an impact on human health, bacterial monitoring is crucial [14]. There is a great concern regarding methicillin-resistant *S. aureus* (MRSA), vancomycin-resistant enterococci (VRE), colistin-resistant *Enterobacteriaceae*, as well as extended-spectrum-lactamase (ESBL) and carbapenemase-producing *Enterobacteriaceae* [15,16,17]. There is no information available on the prevalence of these bacteria in horsemeat. However, ESBL-producing *E. coli* and methicillin-resistant *S. aureus* (MRSA) have been found in horses, and they could be transferred to horsemeat during processing [10,18,19].

The aim of this work was to assess the microbiological safety and quality of horsemeat, including the antimicrobial resistance of relevant bacteria.

## 2. Materials and Methods

### 2.1. Horsemeat Samples and Microbiological Determinations

A total of 19 fresh horsemeat samples were collected in Logroño (Spain) from two hypermarkets (12 samples from A and 7 samples from B) between June and September 2020 (Table S1, Supplementary Materials). They were transferred to the university facilities under refrigeration and analysed as quickly as possible.

For the microbiological analysis, 10 grams were obtained and blended, as described previously [20]. The microbial groups evaluated, as well as the media used and conditions employed, are shown in Table 1. The methodology used has been described previously [20,21,22,23]. Also, *Enterobacteriaceae* colistin resistant was determined in ChromID Colistin agar.

### 2.2. Isolation and Identification

A total of 3 to 5 typical colonies were chosen from each culture medium inoculated with horsemeat samples. Strains were purified and maintained as described previously [20]. Bacterial identification was performed using the MALDI-TOF Biotyper (Bruker, Daltonik, Bremen, Germany).

### 2.3. Phenotypic Antimicrobial Resistance of Y. enterocolitica Isolates

The resistance of *Y. enterocolitica* strains was evaluated, using the disk diffusion method, against the following antibiotics (Oxoid, Basingstoke, Hampshire, UK): cefpodoxime (CPD, 10 µg), cefepime (FEP, 30 µg), ceftriaxone (CRO, 30 µg), cefoxitin (FOX, 30 µg), ceftazidime (CAZ, 30 µg), cefotaxime (CTX, 30 µg), ampicillin (AMP, 10 µg), aztreonam (ATM, 30 µg), ampicillin/surbactam (SAM, 10/10 µg), amoxicillin-clavulanate (AUG, 20/10 µg), imipenem (IPM, 10 µg), ertapenem (ETP, 10 µg), doripenem (DOR, 10 µg), meropenem (MEM, 10 µg), trimethoprim/sulfamethoxazole (SXT, 1.25/23.75 µg), piperacillin (PRL, 100 µg), trimethoprim (W, 5 µg), chloramphenicol (C, 30 µg), sulfadiazine (SUZ, 300 µg), minocycline (MH, 30 µg), tetracycline (TE, 30 µg), doxycycline (DO, 30 µg), tigecycline (TE, 30 µg), levofloxacin (LEV, 5 µg), enrofloxacin (ENR, 5 µg), norfloxacin (NOR, 10 µg), ciprofloxacin (CIP, 5 µg), nalidixic acid (NA, 30 µg), gatifloxacin (GAT, 5 µg), amikacin (AK, 30 µg), kanamycin (K, 30 µg), gentamicin (CN, 10 µg), streptomycin (S, 10 µg), nitrofurantoin (F, 300 µg), and tobramycin (TOB, 10 µg). The strains were classified as resistant, susceptible, or intermediate, according to the CLSI guidelines [24].

### 2.4. Phenotypic Antimicrobial Resistance of Staphylococcus *spp.*

The phenotypic antimicrobial resistance of *Staphylococcus* spp. was evaluated, using the disk diffusion method, against the following antibiotics: cefoxitin (FOX, 30 µg), ceftaroline (CPT, 30 µg), clindamycin (CMN, 2 µg), penicillin (P, 10 UI), fusidic acid (FAD, 10 µg), trimethoprim -sulfamethoxazole (SXT 1.25:23.75 µg), trimethoprim (W, 5 µg), tetracycline (TE, 30 µg), minocycline (MH, 30 µg), enrofloxacin (ENR, 5 µg), ciprofloxacin (CIP, 5 µg), gatifloxacin (GAT, 5 µg), doxycycline (DO, 30 µg), norfloxacin (NOR, 5 µg), levofloxacin (LEV, 5 µg), gentamicin (CN, 10 µg), amikacin (AK, 30 µg), kanamycin (K, 30 µg), erythromycin (ERY, 15 µg), streptomycin (S, 10 UI), tobramycin (TOB,10 µg), sulfadiazine (SUZ, 300 µg), mupirocin (PUM, 200 µg), tylosin (TY, 30 µg), lincomycin (MY, 15 µg), nitrofurantoin (F, 300 µg), chloramphenicol (C, 30 µg), tedizolid (TZD, 2 µg), linezolid (LZD, 30 µg), vancomycin (VA, 30 µg), and rifampicin (RD, 5 µg). The strains were classified as resistant, susceptible, or intermediate, according to the CLSI guidelines [24].

### 2.5. Phenotypic Antimicrobial Resistance of Enterococci

The resistance of enterococci was evaluated, using the disk diffusion method, against the following antibiotics: norfloxacin (NOR, 5 µg), levofloxacin (LEV, 5 µg), enrofloxacin (ENR, 5 µg), ciprofloxacin (CIP, 5 µg), vancomycin (VA, 30 µg), chloramphenicol (C, 30 µg), doxycycline (DO, 30 µg), tetracycline (TE, 30 µg), teicoplanin (TEC, 30 µg), tigecycline (TGC, 15 µg), linezolid (LZD, 30 µg), gentamicin (CN, 120 µg), ampicillin (AMP, 10 µg), nitrofurantoin (F, 300 µg), imipenem (IPM, 5 µg), and minocycline (MH, 30 µg). The strains were classified as resistant, susceptible, or intermediate, according to the CLSI guidelines [24].

### 2.6. Phenotypic Antimicrobial Resistance of L. monocytogenes

The phenotypic antimicrobial resistance of of *L. monocytogenes* was evaluated, using the disk diffusion method, against the following antibiotics: amikacin (AK, 30 µg), streptomycin (S, 10 µg), amoxycillin/clavulanic acid (AUG, 30 µg), gentamicin (CN, 10 µg), ampicillin (AMP, 2 µg), tobramycin (TOB, 10 µg), penicillin G (PNG, 10 µg), cefotaxime (CTX, 30 µg), oxacillin (OX, 1 µg), ceftaroline (CPT, 30 µg), meropenem (MEM, 10 µg), imipenem (IPM, 10 µg), ciprofloxacin (CIP, 5 µg), levofloxacin (LEV, 5 µg), enrofloxacin (ENR, 5 µg), norfloxacin (NOR, 10 µg), minocycline (MH, 30 µg), doxycycline (DO, 30 µµg), tetracycline (TE, 30 µg), teicoplanin (TEC, 30 µg), tigecycline (TGC, 15 µg), chloramphenicol (C, 30 µg), linezolid (LZD, 30 µg), vancomycin (VA, 30 µg), erythromycin (ERY, 15 µg), quinupristin/dalfopristin (QD, 15 µg), nitrofurantoin (F, 300 µg), trimethoprim/sulphamethoxazole 1:19 (SXT, 25 µg), and rifampicin (RD, 5 µg). The strains were classified as resistant, susceptible, or intermediate, according to the CLSI guidelines [24].

### 2.7. Phenotypic Antimicrobial Resistance of Stenotrophomonas maltophilia

The phenotypic antimicrobial resistance of *Stenotrophomonas maltophilia* was evaluated, using the disk diffusion method, against the following antibiotics: ampicillin (AMP, 10 µg), aztreonam (ATM, 30 µg), cefazoline (CZ, 30 µg), cefotaxime (CTX, 30 µg), cefpodoxime (CPD, 10 µg), ceftazidime (CAZ, 30 µg), ciprofloxacin (CIP, 5 µg), chloramphenicol (C, 30 µg), doxycycline (DO, 30 µg), enrofloxacin (ENR, 5 µg), fosfomycin (FOS, 200 µg), gentamicin (CN, 10 µg), imipenem (IPM, 10 µg), levofloxacin (LEV, 5 µg), minocycline (MH, 30 µg), netilmicin (NET, 30 µg), norfloxacin (NOR, 10 µg), piperacillin (PRL, 100 µg), sulfadiazine (SUZ, 300 µg), tetracycline (TE, 30 µg), ticarcillin-clavulanic (TTC, 75/10 µg), tobramycin (TOB, 10 µg), and trimethoprim -sulfamethoxazole (SXT, 1.25:23.75 µg). The strains were classified as resistant, susceptible, or intermediate, according to the CLSI guidelines [24].

### 2.8. Phenotypic Antimicrobial Resistance of Vagococcus fluvialis

The phenotypic antimicrobial resistance of *Vagococcus fluvialis* was evaluated, using the disk diffusion method, against the following antibiotics: amikacin (AK, 30 µg), ampicillin (AMP, 10 µg), ceftriaxone (CRO, 30 µg), ciprofloxacin (CIP, 5 µg), clindamycin (CMN, 30 µg), chloramphenicol (C, 30 µg), doxycycline (DO,30 µg), enrofloxacin (ENR, 5 µg), erythromycin (ERY, 15 µg), streptomycin (S, 300 µg), fosfomycin (FOS, 200 µg), gentamicin (CN, 10 µg), imipenem (IPM, 10 µg), levofloxacin (LEV, 5 µg), linezolid (LZD, 30 µg), minocycline (MH, 30 µg), nitrofurantoin (F, 300 µg), norfloxacin (NOR, 10 µg), oxacillin (OX, 1 µg), penicillin (P, 10 UI), quinupristin/dalfopristin (QD, 15 µg), rifampicin (RD, 5 µg), sulfadiazine (SUZ, 300 µg), teicoplanin (TEC, 30 µg), tetracycline (TE, 30 µg), tigecycline (TGC, 15 µg), tobramycin (TOB, 10 µg), trimethoprim-sulfamethoxazole (SXT, 1.25:23.75 µg), and vancomycin (VA, 30 µg). The strains were classified as resistant, susceptible, or intermediate, according to the CLSI guidelines [24].

### 2.9. Statistical Analysis

The analysis of variance was performed using SPSS version 26 software (IBM SPSS ^S3tatistics^). Tukey’s test for comparison of means was carried out using the same program. The level of significance was determined at *p* < 0.05.

## 3. Results

Table 2 shows the distribution of microbial counts in horsemeat. The mean counts for mesophiles were 4.89 ± 1.08 log CFU/g. Mesophile counts were below 7 log CFU/g, except in one sample from hypermarket B; counts varied between 2.78 and 7.03 log CFU/g. *Pseudomonas* populations under 1 log CFU/g were obtained in 9 horsemeat samples (47.37%). The counts in the other 10 samples varied between 1.30 and 2.84 log CFU/g. *Enterobacteriaceae* populations under 1 log CFU/g were obtained in 12 horsemeat samples (63.16%). The counts in the other 7 samples varied between 1.78 and 4.10 log CFU/g. *Staphylococcus* spp. populations under 1 log CFU/g were obtained in 15 horsemeat samples (78.95%). The counts in the other 4 samples varied between 1.30 and 3.41 log CFU/g. All the samples from hypermarket B showed *Enterococcus* spp. counts below 1 log CFU/g, while 9 of 12 samples from hypermarket A shown counts below this value, and the other 3 samples showed counts between 1.30 and 1.60 log CFU/g. No significant differences (*p* > 0.05) in microbial counts were found between hypermarket A and B.

The counts of *Listeria* spp. and *L. monocytogenes* were below 1 log CFU/g in all the samples. *Listeria* spp. was detected in 6 samples (31.58%), 4 from hypermarket A and 2 from hypermarket B. *L. monocytogenes* was detected in 4 samples (21.05%), 3 from hypermarket A, and 1 from hypermarket B. *L. welshimeri* was isolated in 1 sample from hypermarket A, while *L. innocua* was isolated in 1 sample from hypermarket B. *Campylobacter* spp. was not found in any sample.

Table 3 shows the bacteria isolated in plate count agar (PCA) in samples taken from hypermarket A and B. In both hypermarkets, the predominant group was lactic acid bacteria (42.86–56.67%). In the hypermarket A samples, *C. divergens*, *Lactobacillus* spp., *L. lactis*, and *C. maltaromaticum* were identified. On the other hand, in the hypermarket B samples, *Lactococcus piscium* and *Lactobacillus sakei* were also found. *Brochothrix thermosphacta* was found in both hypermarkets A and B, with prevalence of 10.2% and 3.33%, respectively.

*Pseudomonas* spp. and *Enterobacteriaceae* accounted for 14.28% and 8.16% of isolates from hypermarket A, respectively. In hypermarket B, *Pseudomonas* spp. accounted for 20% of the isolates, while *Enterobacteriaceae* only represented 3.33% of the isolates. *P. fragi*, *P. extremorientalis,* and *S. proteamaculans* were isolated in samples from both hypermarkets. However, the species *P. gessardii*, *P. lundensis*, and *Buttiauxella gaviniae* were only isolated in horsemeat from hypermarket A, while *P. fluorescens* and *P. antarctica* were only found in samples from hypermarket B.

*Acinetobacter* spp., *Chryseobacterium* spp., *Stenotrophomonas* spp., *Kocuria* spp., and *Microbaterium* spp. were isolated in a lesser extent (2.04–4.08%).

**Figure 1:**
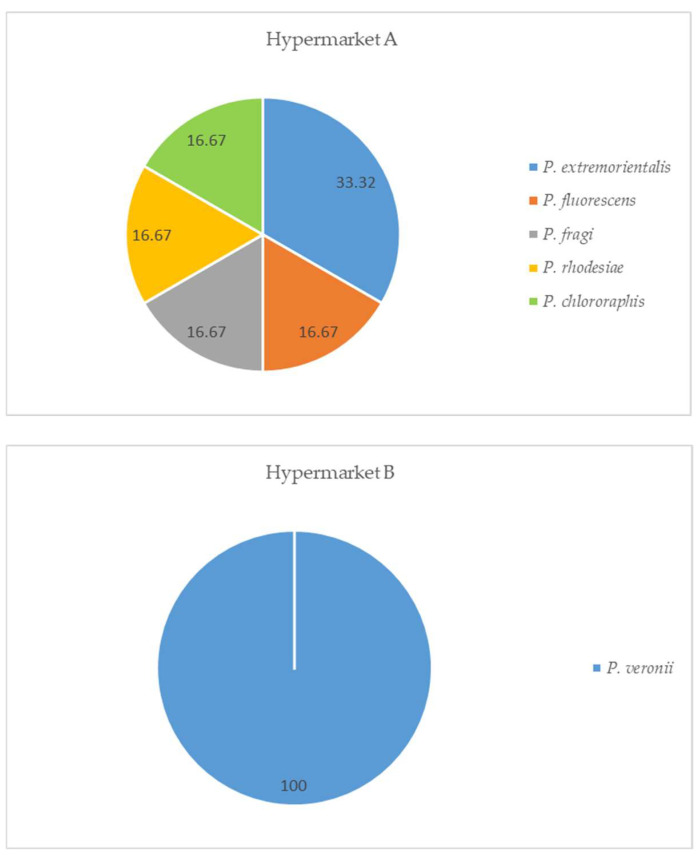
Identification of *Pseudomonas* spp. isolated from fresh horsemeat by place of purchase, in percentages (recovered from medium specific for *Pseudomonas*).

In relation to *Pseudomonas* spp. isolated from specific chromogenic medium for the isolation of this bacterium, five species were identified in hypermarket A: *P. extremorientalis*, *P. fluorescens*, *P. fragi*, *P. rhodesiae,* and *P. chlororaphis*, while in hypermarket B, only *P. veronii* was found (Figure 1).

**Figure 2:**
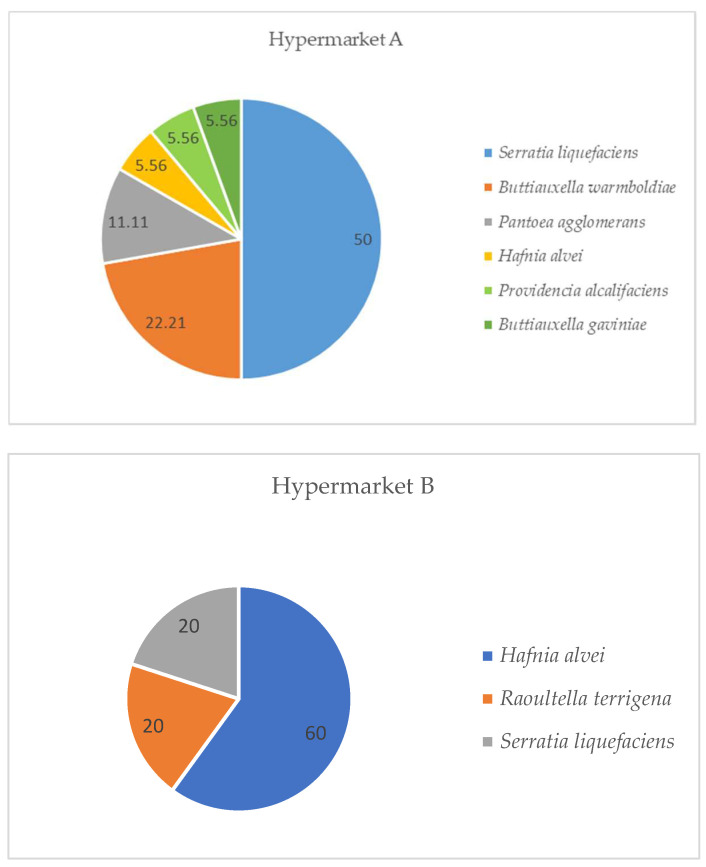
Identification of *Enterobacteriaceae* isolated from fresh horsemeat by place of purchase, in percentages (recovered from McConkey agar).

In relation to *Enterobacteriaceae* in horsemeat, in both hypermarkets *Serratia liquefaciens* and *Hafnia alvei* were identified. In the horsemeat from hypermarket A, the predominant genus was Serratia, with 50% of the isolates. In the case of horsemeat from hypermarket B, *Hafnia alvei* was the predominant species, with 60% of the isolates. The species *B. warmboldiae*, *P. agglomerans*, *P. alcalifaciens*, and *B. gaviniae* were found exclusively in hypermarket A (5.56–22.21%), while *R. terrigena* was isolated from samples of hypermarket B (20%). *E. coli* was not isolated in any sample (Figure 2).

When the medium ChromID ESBL was used, *E. coli* was not isolated. When using ChromID CARBA, *Enterobacteriaceae* was not isolated, but *Stenotrophomonas maltophilia* was found in one sample from hypermarket A. This bacterium was also isolated from PCA in the same sample. These two strains of *Stenotrophomonas maltophilia* showed resistance against ampicillin, aztreonam, cefotaxime, cefpodoxime, cefazolin, imipenem, and piperacillin (Table 4).

When using ChromID colistin, *Yersinia enterocolitica* was isolated in one sample from hypermarket A. This strain showed resistance against 12 antibiotics: amoxicillin/clavulanic, ampicillin, ampicillin/sulbactan, aztreonan, cefepime, cefotaxime, cefoxitin, cefpodoxime, ceftriaxone, nalidixic acid, nitrofurantoin, and trimethoprim (Table 4).

**Figure 3:**
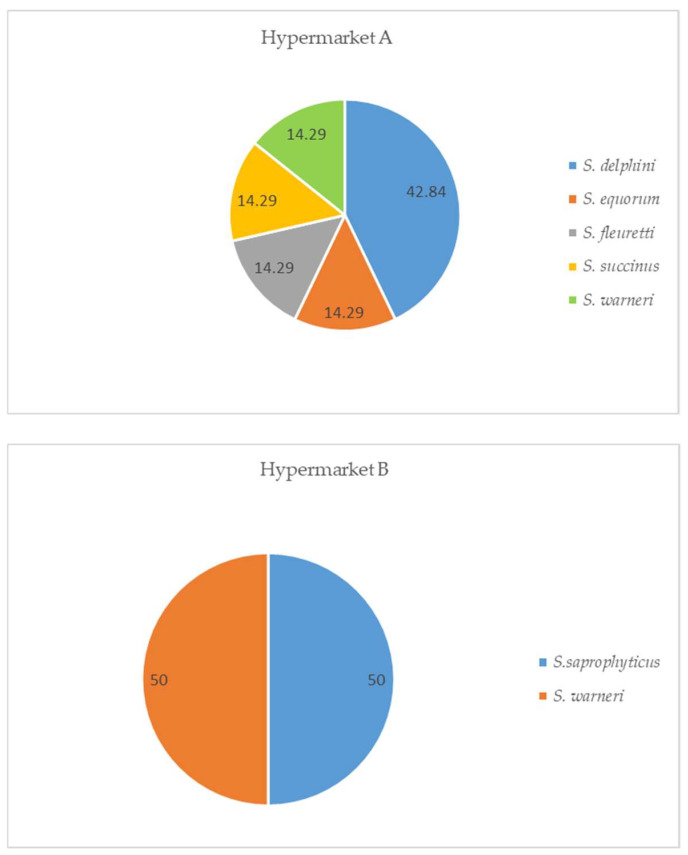
Identification of *Staphylococcus* spp. isolated from fresh horsemeat according to place of purchase, in percentages (recovered from MSA).

Figure 3 shows the *Staphylococcus* spp. identified in horsemeat samples. The species isolated most often from hypermarket A was *S. delphini* (42.84%), while in hypermarket B, the most often identified species were *S. saprophyticus* (50%) and *S. warneri* (50%). *S. equorum*, *S. fleurettii*, *S. succinus*, and *S. warneri* were also isolated from hypermarket A.

All of the isolated *Staphylococcus* spp. strains were susceptible to all the antibiotics assayed, except for two. One strain of *S. delphini* showed resistance against mupirocin and cefoxitin, while one strain of *S. saprophyticus* showed resistance against doxycycline, penicillin, and tetracycline (Table 4). When using ChromID MRSA, *S. aureus* was not isolated. However, *V. fluvialis* was isolated in a sample from hypermarket A. This strain showed resistance against clindamycin, linezolid, nitrofurantoin, oxacillin, penicillin, quinupristin/dalfopristin, teicoplanin, and vancomycin (Table 4).

*Enterococcus malodoratus* was the only enterococci isolated from horsemeat that was susceptible to all the antibiotics tested (Table 4). None enterococci was isolated from ChromID VRE agar.

All of the isolated *L. monocytogenes* strains were resistant to ampicillin, cefotaxime, and oxacillin. Resistance against bezilpenicillin, meropenem, and nitrofurantoin was found in 75%, 50%, and 25% of the strains isolated, respectively (Table 4).

## 4. Discussion

The mean counts of mesophiles obtained in this work (4.89 ± 1.08 log UFC/g) were higher than those reported by Pavlidis et al. and Gomez and Lorenzo (2.77 ± 0.10 log CFU/g and 4.13 log CFU/g, respectively) [6,25]. Higher pseudomonas counts have been reported by Pavlidis et al. (2021) (2.72 ± 0.17 log10 CFU/g) and Lorenzo and Gomez (4.24 log10 CFU/g) [6,8].

As noted in the current work, other researchers have pointed out that the dominant bacteria in horsemeat from Belgian markets were lactic acid bacteria [7]. In contrast, other authors observed that the dominant bacteria in samples from the European market were pseudomonads, followed by *B. thermosphacta* [6]. These differences can be explained by the storage temperature and the environmental packaging conditions [8,26]. Also, Geeraerts et al. found that *Carnobacterium divergens* and *Lactobacillus* spp. were the dominant lactic acid bacteria in fresh horsemeat [5]. *Lactobacillus sakei*, *Lactococcus piscium*, and *B. thermosphacta* have been also identified by other authors in fresh horsemeat [7]. However, these authors did not isolate *C. maltaromaticum* from fresh horsemeat [7].

The presence of *Pseudomonas* spp. is associated with the spoilage of fresh meat, as it is able to develop under non-aerobic conditions [27]. In this study, the species *P. fragi*, *P. extremorientalis*, *P. gessardii*, *P. fluorescens*, *P. antárctica*, *P. rhodesiae, P. chlororaphis*, and *P. veronii* were identified. These species are frequently found in meat from other animals, as well as on surfaces where meat is handled, some of them being able to form biofilms [28,29].

We also isolated *Chryseobacterium* spp.; this microorganism has been found in the reproductive system of horses [30].

We only found *Enterobacteriaceae* counts above 1 log CFU/g in seven samples (36.84%), six from hypermarket A (50%) and one from hypermarket B (14.29%). These results agree with those reported by Geeraerts et al., who only detected *Enterobacteriaceae* in 11.76% of the samples analysed from the Belgian market, with *Hafnia alvei* being the only species isolated [7]. In contrast, Furuhata et al. found *Enterobacteriaceae* in 93.8% of the horsemeat samples from Japan [9]. Furuhata et al. pointed out that the dominant bacterium was *Hafnia alvei* (19.8%), as we observed in samples from hypermarket B [9]. These authors identified 14 *Enterobacteiaceae* species, while we only identified 9 different species. As did Furuhata et al., we also isolated *Raultella terrigena* and *Serratia liquefaciens* from horsemeat [9]. However, we did not isolate *Klebsiella pneuminiae*, *Enterobacte cloacae*, *Pantoea* spp., *Enterobacter ammigenus, Klebsiella oxytoca*, *Citrobacter yungae*, *E. coli*, or *Proteus mirabilis*. However, we found other species, such as *Serratia liquefaciens*, *Buttiauxella warmboldiae*, *Pantoea agglomerans*, *Providencia alcalifaciens*, *Buttiauxella gaviniae*, *Serratia proteamaculans*, and *Yersinia enterocolitica*. We did not detect *E. coli* in any sample. The differences found could be explained by the varying hygienic conditions, since *Enterobacteriaceae* are considered as a hygiene indicator associated with faecal contamination [31]. *E. coli* has been isolated in faecal samples from horses, and faecal meat contamination is possible during slaughter [31].

Other authors have pointed out that contamination of horsemeat with *Yersinia enterocolitica* could be frequent, since this bacterium is often found in horse faecal samples, and contamination of meat could occur [12]. As in the present work, Seekamela et al. also found *Y. enterocolitica* strains resistant to ampicillin that were isolated from beef and pork meat in South Africa [32]. However, we did not find resistance against tetracycline, chloramphenicol, aztreonam, imipenem, gentamycin, piperacillin, or amikacin. In agreement with the results of Seekamela et al., we did not find resistance against trimethoprim-sulphamethoxazole [32]. We did observe resistance against amoxicillin/clavulanic, ampicillin/surbactam, cefepime, cefotaxime, cefoxitin, cefpodoxime, ceftriaxone, nalidixic acid, and nitrofurantoin. Terentjeva et al. found resistance to ampicillin, but not to cefotaxime and trimethoprim, in *Y. enterocolitica* strains isolated from beef and pork in Latvia [33]. Resistance to amoxicillin/clavulanic has also been reported in *Y. enterocolitica* strains isolated from beef and poultry. It should be note that we found resistance to aztreonam, an antibiotic that is categorized in “Category A: antimicrobial to avoid” for animals [34].

We only isolated *Staphylococcus* spp. from four samples (21.05%). Other authors have pointed out that the dominant example of *Staphylococcus* spp. in fresh horsemeat from the Belgian market is *S. equorum*, instead of *S. delphini* and *S. saprophyticus*, as was observed in the present work [7]. Geeraerts et al. isolated *S. simulans* and *S. xylosus*, species that were not found in the present work [7]. However, we isolated *delphini*, *S. fleurettii*, *S. succinus*, and *S. warneri. S. delphini* and *S equorum* have been isolated from the skin of horses, and in consequence, they could contaminate meat during processing [35,36,37]. We did not isolate *S. aureus* from horsemeat, although other authors have pointed out that horses are a reservoir of this microorganism, and MRSA strains have even been isolated from horse [13,18,38,39].

Low resistance rates were found in the *Staphylococcus* spp. strains. We only observed one strain of *S. delphini* that showed resistance against mupirocin and cefoxitin, while one strain of *S. saprophyticus* showed resistance against doxycycline, penicillin, and tetracycline. Penicillin is one of the most common antibiotics used in the treatment of horse infections and in consequence, resistance to this antibiotic could be found [30]. The resistance to tetracyclines has also been reported by other authors in staphylococci strains isolated from horses in France and Canada [40,41]. It should be noted that we found resistance to mupirocin, an antibiotic that is categorized in “Category A: antimicrobial to avoid” for animals [34].

Low enterococci levels were found in the present work, and enterococci was identified in only 15.79% of the samples. Higher percentages of samples showing the presence of enterococci have been reported in goat, pork, and poultry meat [20,42]. The only species found in the present work was *E. malodoratus*, and this isolated strain showed susceptibility to all the antibiotics tested.

A lower prevalence of *Listeria* spp. and *L monocytogenes* has been reported by Assis et al. in horsemeat from Brazil (18.2% and 7.4%, respectively, versus 31.58% and 21.05% found in the present work) [11]. Also, a lower prevalence of *L monocytogenes* has been reported in poultry and pork meat [43,44,45]. In the current study, all the samples showed counts below 2 log CFU/g. High resistance rates of *L. monocytogenes* against ampicillin, cefotaxime, and oxacillin were observed (100%). Other authors have also found resistance against oxacillin and ampicillin, cefotaxime, meropenem, benzylpenicillin, and nitrofurantoin in *L. monocytogenes* strains isolated from beef, pork, and poultry meat in Spain and China [43,46]. It should be taken into account that ampicillin and benzylpenicillin are commonly used in the treatment of infections caused by *L. monocytogenes* [47]. It should also be noted that we found resistance to aztreonam, which is categorized in “Category A: antimicrobial to avoid” for animals [34].

As in the present work Collobert et al. did not isolate *Campylobacter* spp. from horse carcasses [48]. Several studies indicate that the presence of *Campylobacter* spp. in horse faeces is low; consequently, the contamination of horsemeat during processing is low compared to that of poultry meat [49].

*Stenotrophomonas maltophilia* is considered as an emerging Gram-negative multi-resistant bacterium [50,51]. This microorganism has been associated with respiratory and urinary human infections, as well as respiratory infections in horses [51,52]. *S. maltophilia* has been isolated from goat, rabbit, and poultry meat [53,54,55], but there is no information available regarding its presence in horsemeat. However, *S. maltophilia* has been detected in horse manure from France and Tunisia, and the majority of strains isolated were resistant to 7–9 antibiotics [56]. The high rates of antibiotic resistance in strains isolated from horse manure has been associated with antibiotic presence, since animals are often treated with antibiotics [56]. In the present study, *S. maltophilia* showed resistance against seven antibiotics: ampicillin, aztreonam, cefotaxime, cefpodoxime, cefazolin, imipenem, and piperacillin. The high resistance rates of *S. maltophilia* against ceftazidime and imipenem has also been observed in strains isolated from horse manure in France and Tunisia [56]. It should be highlighted the resistance found against aztreonam, which has been recommended for the treatment of infections caused be this microorganism, is categorized in “Category A: antimicrobial to avoid” for animals [34,57].

*V. fluvialis* has been isolated from beef, pork, and chicken meat [58,59,60,61,62]. The *Vagococcus* microorganism is considered as an emerging pathogen in humans [63,64,65]. *V. fluvvialis* infections could be underestimated, since this bacterium is frequently improperly identified as *Enterococcus* spp. [65]. The identification by means of MALDI-TOF could help to detect this bacterium. Most of the studies on *V. fluvialis* antibiotic resistance have been carried out using strains isolated from animals or from human infections. There is not data available on *V. fluvialis* isolated from meat. In the present work, the *V. fluvialis* strain showed resistance against clindamycin, linezolid, nitrofurantoin, oxacillin, penicillin, quinupristin/dalfopristin, teicoplanin, and vancomycin. Also, Matajira et al. and Texiera et al. reported that *V. fluvialis* isolated from pigs in Brazil showed resistance to clindamycin [61,66]. According to Rancero et al., the most active antibiotics against *V. fluvialis* isolated from human infection were vancomycin, ampicillin, trimethoprim/sulfamethoxazole, linezolid, and teicoplanin, while resistance to fluoroquinolones and tetracyclines was observed in 40 and 80% of the strains, respectively [65]. We observed resistance to vancomycin, teicoplanin, and linezolid. Other studies on strains isolated form human infections reported resistance to trimethoprim/sulfamethoxazole and levofloxacin, while susceptibility to ampicillin, minocycline, vancomycin, and linezolid was observed [63]. Other authors have also reported resistance to clindamycin in strains isolated from human infections [66]. Chen et al. reported susceptibility to tigecycline, vancomycin, quinupristin/dalfopristin, and linezolid in human infection isolates, and moderate sensitivity to ciprofloxacin, levofloxacin, ampicillin/sulbactam, erythromycin, and tetracycline [65]. It should be note that we found resistance to linezolid and vancomycin, antibiotics that are categorized in “Category A: antimicrobial to avoid” for animals [34].

## 5. Conclusions

The present work advises that horsemeat could be a source of both emerging (*V. fluvialis* and *S. maltophilia*) and recognised foodborne pathogens (*L. monocytogenes* and *Y. enterocolitica*). Moreover, horsemeat can be a source of multi-resistant bacteria. The presence of multi-resistant *Y. enterocolitica*, *V. fluvialis*, and *S. maltophilia* in horsemeat is of great concern, and special actions to reduce meat contamination should be adopted in the framework of the One Health approach. Adequate processing, handling, cleaning, and disinfecting procedures could help to avoid cross-contamination. Resistance to critical antibiotics, according to the European Medicine Agency (EMA) standards, including mupirocin, aztreonam, linezolid, and vancomycin, was observed in strains isolated from horsemeat.

## Figures and Tables

**Table 1:** Media and conditions used for microbiological evaluation.

Culture Media	Microbial Group	Incubation Time (h)	Incubation Temperature (°C)
Plate Count Agar	Mesophiles	48	30
Chromogenic Agar for Pseudomonas	Pseudomonas	72	30
MacConkey Agar	*Enterobacteriaceae*	24	37
Mannitol Salt Agar	Staphylococci	36	35
Kanamycin Esculin Azide Agar	Enterococci	48	37
ALOA Agar	*Listeria* spp. and *L. monocytogenes*	24	30
Brilliance Campy Count Agar ^1^	*Campylobacter* spp.	48	42
ChromID ESBL Agar	ESBL-producing *E. coli*	24	37
ChromID CARBA Agar	*Enterobacteriaceae* carbapenemase-producers	24	37
ChromID Colistin Agar	Colistin-resistant *Enterobacteriaceae*	24	37
ChromID VRE Agar	Vancomycin-resistant enterococci	24	37
ChromID MRSA Agar	Methicillin-resistant *S. aureus*	24	37

^1^ incubated under microaerobic conditions.

**Table 2:** Distribution of microbial counts in fresh horsemeat (log CFU/g).

Microbial Group	Place of Purchase	Distribution of Microbial Counts ^2^			Minimum Counts	Maximum Counts	Means± Standard Deviation ^4^
Mesophiles		<1 ^3^	1–7 ^3^	>7 ^3^			
	A (*n* ^1^ = 12)	0	12	0	3.05	6.30	4.92 ± 1.06 ^A 5^
	B (*n* = 7)	0	6	1	2.78	7.03	4.84 ± 1.13 ^A^
	A + B (*n* = 19)	0	18	1	2.78	7.03	4.89 ± 1.08
*Pseudomonas* spp.		<1 ^3^	1–6 ^3^	>6 ^3^			
	A (*n* = 12)	5	7	0	1.30	2.84	2.18 ± 0.00 ^A^
	B (*n* = 7)	4	3	0	2.00	2.47	2.26 ± 0.70 ^A^
	A + B (*n* = 19)	9	10	0	1.30	2.84	2.20 ± 0.40
*Enterobacteriaceae*		<1 ^3^	1–4 ^3^	>4 ^3^			
	A (*n* = 12)	6	6	0	1.78	3.83	2.31 ± 0.79 ^A^
	B (*n* = 7)	6	0	1	4.10	4.10	4.10 ± 0.00 ^A^
	A + B (*n* = 19)	12	6	1	1.78	4.10	2.56 ± 0.97
*Staphylococcus* spp.		<1 ^3^	1–4 ^3^	>4 ^3^			
	A (*n* = 12)	9	3	0	1.30	3.41	2.32 ± 0.30 ^A^
	B (*n* = 7)	6	1	0	1.60	1.60	1.60 ± 0.00 ^A^
	A + B (*n* = 19)	15	4	0	1.30	3.41	2.14 ± 0.69
*Enterococcus* spp.		<1 ^3^	1–3 ^3^	>3 ^3^			
	A (*n* = 12)	9	3	0	1.30	1.60	1.40 ± 0.13
	B (*n* = 7)	7	0	0	-	-	-
	A + B (*n* = 19)	16	3	0	1.30	1.60	1.40 ± 0.13

^1^ *n*, number of samples. ^2^ Data in the table indicate the number of samples presenting the counts indicated (log CFU/g). ^3^ range of counts in log CFU/g). ^4^ means ± standard deviation of samples with counts. ^5^ For each microbial group, means in the same column, not followed by the same letter (subscript), are significantly different (*p* < 0.05).

**Table 3:** Bacteria identified in horsemeat isolated from plate count agar, according to the place of purchase.

Place of Purchase	Microbial Group	Percentage (%)	Species	Percentage (%)
	*Brochothrix* sp.	10.20	*Brochothrix thermosphacta*	10.20
A	Lactic acid bacteria	42.86	*Carnobacterium divergens*	18.37
			*Lactobacillus* sp.	16.33
			*Lactococcus lactis*	4.08
			*Carnobacterium maltaromaticum*	4.08
	*Pseudomonas* spp.	14.28	*P. fragi*	8.16
			*P. extremorientalis*	2.04
			*P. gessardii*	2.04
			*P. lundensis*	2.04
	*Enterobacteriaceae*	8.16	*Serratia proteamaculans*	6.12
			*Buttiauxella gaviniae*	2.04
	*Micrococcaceae*	2.04	*Staphylococcus saprophyticus*	2.04
	Other Gram-negative bacteria	16.33	*Acinetobacter guillouiae*	4.08
			*Chryseobacterium scophthalmum*	4.08
			*Acinetobacter harbinensis*	2.04
			*Chryseobacterium indologenes*	2.04
			*Stenotrophomonas rhizophila*	2.04
			*Stenotrophomonas maltophilia*	2.04
B	*Brochothrix* sp.	3.33	*Brochothrix thermosphacta*	3.33
	Lactic acid bacteria	56.67	*Carnobacterium divergens*	16.67
			*Lactobacillus* sp.	16.67
			*Carnobacterium maltaromaticum*	10.00
			*Lactococcus lactis*	6.67
			*Lactococcus piscium*	3.33
			*Lactobacillus sakei*	3.33
	*Pseudomonas* spp.	20.00	*P. fragi*	6.67
			*P. fluorescens*	6.67
			*P. antarctica*	3.33
			*P. extremorientalis*	3.33
	*Enterobacteriaceae*	3.33	*Serratia proteamaculans*	3.33
	*Micrococcaceae*	6.66	*Staphylococcus warneri*	3.33
			*Kocuria rhizophila*	3.33
	Other Gram-negative bacteria	9.99	*Chryseobacterium indologenes*	3.33
			*Chryseobacterium shigense*	3.33
			*Microbacterium liquefaciens*	3.33

**Table 4:** Antimicrobial resistance phenotypes of bacteria isolated from horsemeat.

*Species*	Antibiotic Resistance Phenotype ^1^	Sample ^2^	Place of Purchase
*S. maltophilia*	AMP-ATM-CTX-CPD-CZ-IPM-PRL	H11(PCA)	A
		H11 (CARB)	A
*Y. enterocolitica*	AUG-AMP-SAM-ATM-FEP-CTX-FOX-CPD-CRO-NA-F-W	H07 (COL)	A
*S. delphini*	PUM-FOX	H02 (MSA)	A
	susceptible to all antibiotics tested	H03 (MSA)	A
*S. saprophyticus*	DO-P-TE	H06 (MSA)	B
	P	H11 (PCA)	A
*S. warneri*	susceptible to all antibiotics tested	H03 (MSA)	A
		H06(PCA)	B
		H06 (MSA)	B
*S. fleuretti*	susceptible to all antibiotics tested	H02 (MSA)	A
*S. succinus*	susceptible to all antibiotics tested	H06 (MSA)	A
*S. equorum*	susceptible to all antibiotics tested	H05 (MSA)	A
*E. maldoratus*	susceptible to all antibiotics tested	H13 (KN)	A
*V. fluvialis*	CMN-LZD-F-OX-P-QD-TEC-VA	H14 (MRSA)	A
*L. monocytogenes*	AMP-PNG-CTX-MEM-OX	H13 (ALOA)	A
	AMP-PNG-CTX-MEM-OX	H14 (ALOA)	A
	CTX-F-OX	H11 (ALOA)	B
	AMP-PNG-CTX-OX	H16 (ALOA)	A

^1^ AMP: ampicillin, ATM: aztreonan, CTX: cefotaxime, CPD: cefpodoxime, CZ: cefazoline, IPM: imipenem, PRL: piperacillin, AUG: amoxicillin-clavulanate, SAM: ampicillin/surbactam, FEP: cefepime, FOX: cefoxitin, CRO: ceftriaxone, NA: nalidixic acid, F: nitrofurantoin, W: trimethoprim, DO: doxycycline, PUM: mupirocin, P: penicillin, TE: tetracycline, CMN: clindamycin, LZD: linezolid, OX: oxacillin, QD: quinupristin/dalfopristin, TEC: teicoplanin, VA: vancomycin, PNG: bezilpenicillia, MEM: meropenem. ^2^ isolation medium. PCA: plate count agar, CARB: ChromID CARBA agar, COL: ChromID Colistin agar, MSA: Mannitol salt agar, KN: Kanamycin Esculin Azide agar, MRSA: ChromID MRSA Agar, ALOA: ALOA agar.

## Data Availability

The raw data supporting the conclusions of this article will be made available by the authors on request.

## Supplementary Materials

The following supporting information can be downloaded at: https://www.mdpi.com/article/10.3390/microorganisms12091775/s1, Table S1: Samples taken by place of purchase.
