# Supplementary material for: Microbiological Quality and Antibiotic Resistance of Relevant Bacteria from Horsemeat
Source: Microorganisms. 2024 Aug 28;12(9):1775. doi: 10.3390/microorganisms12091775 (PMC11433819; doi:10.3390/microorganisms12091775)
Supplement: Supplementary file 1 [file microorganisms-12-01775-s001.zip › microorganisms-3175844-supplementary.pdf]

Table S1. Samples taken by place of purchase.

Place of purchase	Sample
Hypermarket A	H01
	H02
	H03
	H05
	H07
	H09
	H10
	H11
	H13
	H14
	H17
	H19
Hypermarket B	H04
	H06
	H08
	H12
	H15
	H16
	H18
